# Age‐specific impacts of vegetation functional traits on gastrointestinal nematode parasite burdens in a large herbivore

**DOI:** 10.1111/1365-2656.13978

**Published:** 2023-07-05

**Authors:** Ellis Wiersma, Robin J. Pakeman, Xavier Bal, Jill G. Pilkington, Josephine M. Pemberton, Daniel H. Nussey, Amy R. Sweeny

**Affiliations:** ^1^ Institute of Ecology & Evolution, School of Biological Science University of Edinburgh Edinburgh UK; ^2^ James Hutton Institute Aberdeen UK; ^3^ School of Biosciences University of Sheffield Sheffield UK

**Keywords:** grazing quality, helminth, home range, host–parasite dynamics, long‐term study, Soay sheep, strongyle

## Abstract

1. Gastrointestinal nematode (GIN) parasites play an important role in the ecological dynamics of many animal populations. Recent studies suggest that fine‐scale spatial variation in GIN infection dynamics is important in wildlife systems, but the environmental drivers underlying this variation remain poorly understood.
2. We used data from over two decades of GIN parasite egg counts, host space use, and spatial vegetation data from a long‐term study of Soay sheep on St Kilda to test how spatial autocorrelation and vegetation in an individual's home range predict parasite burden across three age groups. We developed a novel approach to quantify the plant functional traits present in a home range to describe the quality of vegetation present.
3. Effects of vegetation and space varied between age classes. In immature lambs, strongyle parasite faecal egg counts (FEC) were spatially structured, being highest in the north and south of our study area. Independent of host body weight and spatial autocorrelation, plant functional traits predicted parasite egg counts. Higher egg counts were associated with more digestible and preferred plant functional traits, suggesting the association could be driven by host density and habitat preference.
4. In contrast, we found no evidence that parasite FEC were related to plant functional traits in the host home range in yearlings or adult sheep. Adult FEC were spatially structured, with highest burdens in the north‐east of our study area, while yearling FEC showed no evidence of spatial structuring.
5. Parasite burdens in immature individuals appear more readily influenced by fine‐scale spatial variation in the environment, highlighting the importance of such heterogeneity for our understanding of wildlife epidemiology and health. Our findings support the importance of fine‐scale environmental variation for wildlife disease ecology and provides new evidence that such effects may vary across demographic groups within a population.

## Graphical abstract

By developing a new method for linking plant community ecology with disease measures, this paper provides additional evidence that fine‐scale environmental variation can have varying effects on different demographic groups. This paper also consider spatial autocorrelation in its analyses, an important and often missed aspect of disease studies.

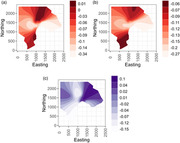

## 1 INTRODUCTION

Gastrointestinal nematode (GIN) parasites play an important role in the ecology of wildlife populations, harming the host, and impacting individual fitness and population dynamics (Coulson et al., 2018). Studies in livestock systems provide detailed understanding of the factors influencing GIN transmission and the development and maintenance of immunity under relatively controlled environmental conditions (Roeber et al., 2013; Smith et al., 2009; Vlassoff et al., 2001), but without incorporating the complexity evident in wild systems. Host factors such as age, sex and body condition are well‐established predictors of GIN burdens in wildlife systems (Lynsdale et al., 2017). However, recent studies have identified complex fine‐scale patterns of spatial and temporal variation in parasite burden over and above the effects of these host variables (Albery et al., 2019, 2020; Sweeny, Albery, et al., 2021; Verheyden et al., 2020). While variation in host habitat use is one potential explanation for spatial variation in parasite burdens in the wild (e.g. Carbayo et al., 2019), our understanding of the factors regulating variation in GIN parasite burdens in natural populations experiencing complex spatially and temporally variable environmental conditions remains limited, representing an important gap in our understanding of wildlife disease epidemiology.

Transmission of GIN is driven both by host factors influencing susceptibility and by environmental factors influencing exposure. Most GIN have a lifecycle involving adult males and females living and mating in host guts, producing eggs that are shed via the faeces into the host's environment. These eggs develop into free‐living larval stages, which are ingested by new hosts and then develop into adults (Morand et al., 2007). Both the rate of egg shedding by adult females and the ingestion of infectious larvae by susceptible hosts via contaminated food or water work in tandem to drive helminth dynamics (Brooker et al., 2006; Morand et al., 2007). Furthermore, environmental conditions can influence infectious life stages of GIN outside the host, as well as resource availability and host nutritional state, both of which may be altered over space and time (Becker et al., 2020). Incorporating measures of host and parasite habitat can offer additional insights into complex exposure and susceptibility drivers of host–parasite dynamics varying over spatial and temporal scales.

Herbivore grazing strategies and the faecal–oral transmission of GIN makes herbivores especially vulnerable to spatial variation in fine‐scale habitat and vegetation quality effects on GIN risk and burden. Vegetation type impacts the decomposition of herbivore faeces (Williams & Warren, 2004), and GIN eggs are sensitive to decomposition rates (Nielsen et al., 2007), suggesting that the structure and type of vegetation can directly impact GIN survival and hence, generate strong spatial structure of transmission risk. For example, taller grass swards can provide more stable microclimates for GIN eggs and larvae (Hutchings et al., 2002), potentially retaining moisture and reducing rates of faecal breakdown. Indirectly, vegetation quality may shape host condition, immunity status and ability to resist infection (Budischak et al., 2018) or avoid exposure (Hutchings et al., 2001). For example, female red deer with higher quality home ranges show higher survival probability (Froy et al., 2018), and wild bovids with lower‐quality diets have more GI parasites during drought years (Ezenwa, 2004). However, areas of highest quality grazing may be associated with higher transmission risk, as high‐quality habitats are likely subjected to increased competition for forage and higher densities of individual grazers. This could counter any beneficial effects on host condition associated with high‐quality vegetation and increase transmission due to high levels of GIN exposure. While recent field studies of GIN infection dynamics have detected and accounted for spatial autocorrelation in parasite burden (e.g. red deer, Albery et al., 2018), our understanding of the environmental drivers of spatial variation is limited. Here, we take a novel functional trait‐based approach to characterise the vegetation within the home ranges of individuals to test the extent to which fine‐scale variation in host habitat may shape GIN dynamics.

Describing herbivore habitats in terms of plant species' functional traits—their morphological, physiological and phenological features—can quantify plant species' contribution to ecosystem processes (Roscher et al., 2012; Tilman, 2001) and offer a useful way of making broad ecological predictions without reference to specific species. The mass‐ratio hypothesis states that the dominance and prevalence of certain traits largely determines an ecosystem's functioning (Díaz et al., 2007; Grime, 1998); effectively, the contribution of a species to ecosystem function depends on its trait values and its contribution to biomass in that trophic level. Using a trait‐based approach allows variation in species' identities between systems to be standardised (Weiss & Ray, 2019) and provides a predictive framework within which to work (McGill et al., 2006). For example, both specific leaf area (SLA; Garnier et al., 2004) and leaf dry matter content (LDMC; Pakeman, 2014) have been used as a proxy for productivity. Quantifying the functional traits of plants present within a host's home range can improve understanding of how habitat and food quality influences parasite burdens in wildlife.

The Soay sheep *Ovis aries* (L.) of St. Kilda are a valuable study system to test how habitat quality influences parasite burdens, due to long‐term data on individual space use, GIN burden, and morphometric measurements (Wilson et al., 2004). These sheep are hosts to a range of GIN parasites, predominantly *Teladorsagia circumcincta*, *Trichostrongylus axei* and *Trichostrongylus vitrinus* (Craig et al., 2006), and parasite burdens can be estimated using standard faecal egg counting (FEC) techniques (Cabaret et al., 1998). Previous studies of the Soay sheep have established that heavy GIN burdens are associated with gastrointestinal damage and higher over‐winter mortality risk (Gulland, 1992) and with negative fitness consequences in lambs (Hayward et al., 2011). FEC varies with age: it is highest in immunologically naïve lambs, declines and stabilises at prime age (2–5 years) as immunity to GIN develops, and then increases again in later adulthood (6 years onwards) as immunity wanes (Hayward et al., 2009). Males tend to have higher burdens than females (Wilson et al., 2004). Recently, a detailed map of the spatial distribution of plant species within our study area was used to show that the proportion of *Holcus lanatus*—a common, comparatively high‐quality, easily digestible grass species—cover in an individual's home range predicted lifetime breeding success (Regan et al., 2016).

In this study, we aimed to determine if the vegetation functional traits within an individual's home range were predictive of variation in GIN burdens (measured as FEC) and whether associations are age dependent. We controlled for host weight and spatial patterns of parasite infection to detect effects of vegetation above and beyond host position in space and to rule out indirect effects on condition. We predicted that individuals whose home range contained more digestible and nutritive leaves would have higher GIN burdens as they are associated with more preferred vegetation and hence higher faecal inputs. We also expected taller vegetation, greater leaf size and species indicative of high moisture conditions to have higher FEC through enhanced parasite survival in their free‐living stages. As lambs tend to have little acquired immunity, we expected lamb FEC to be more sensitive to vegetation traits and environmental influences than adults.

## 2 MATERIALS AND METHODS

### 2.1 Study system

**FIGURE 1:**
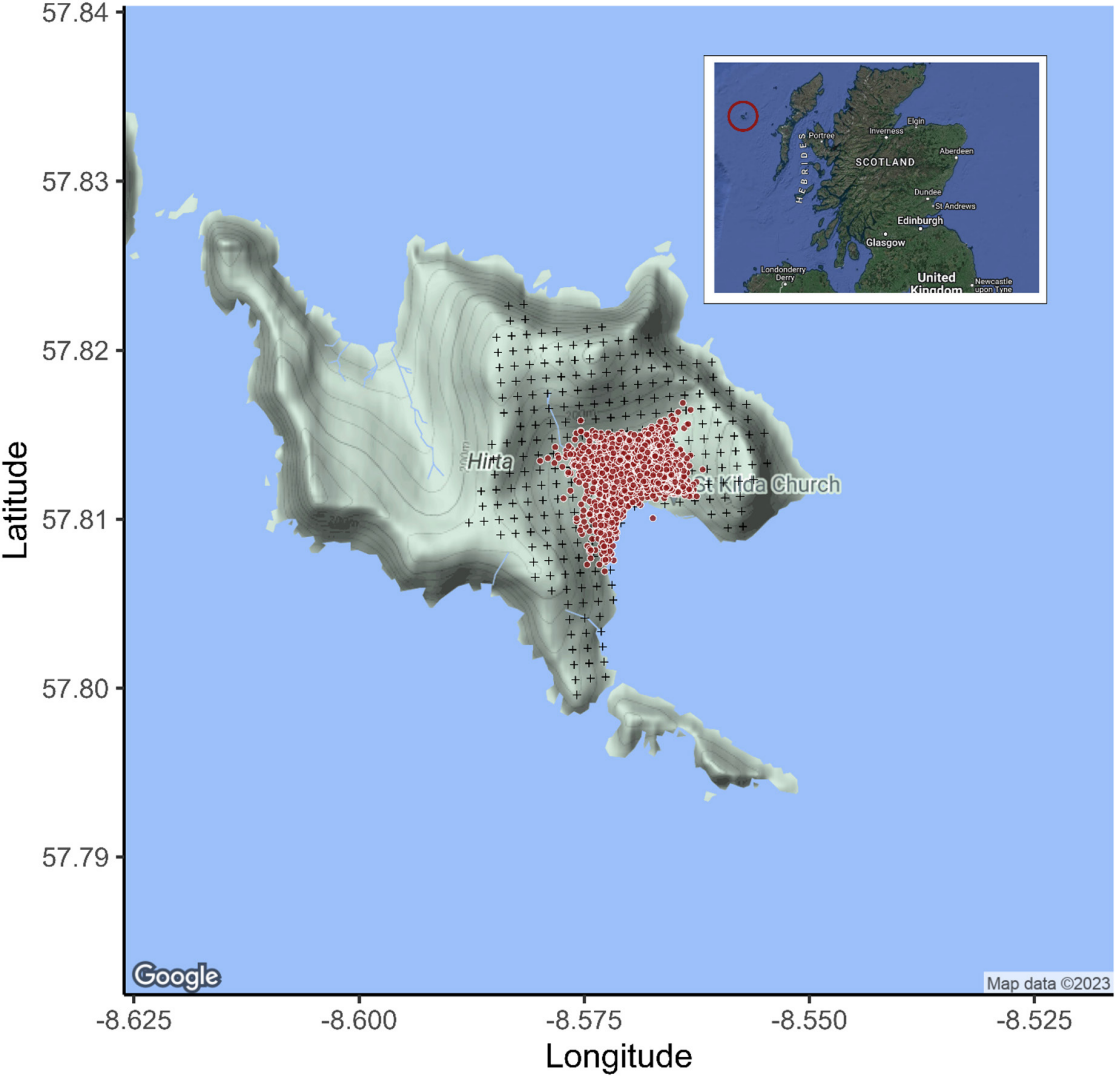
Map of St. Kilda with Village Bay in the southeast region of the island. Hectares used in vegetation surveys and censusing marked by grid. Dots indicate average census location of individual sheep as a relative representation of sheep home ranges.

The Soay sheep of the St. Kilda archipelago (57°49′N, 08°34′W, 65 km NW of the Outer Hebrides, Scotland; Figure 1) are an isolated and unmanaged population (Clutton‐Brock et al., 2004). Individuals in the Village Bay area of the largest island, Hirta, have been individually marked and monitored since 1985 with over 95% of individuals in this area being individually marked at any time (Clutton‐Brock et al., 1992). Each year, lambs are caught shortly after birth in April (typically within a week), marked with unique ear tags and weighed. In August, as many individuals as possible are caught in corral traps over a 2‐week period. Typically, 50%–60% of the resident Village Bay sheep population is captured each August (Clutton‐Brock & Pemberton, 2004) and morphometric measurements are taken, including body mass (to the nearest 100 g). August body mass is an individually repeatable and heritable trait, which is negatively associated with August FEC and positively associated with survival in the following winter (Clutton‐Brock et al., 1992; Craig et al., 2008). All fieldwork was conducted in accordance with the Animals (Scientific Procedures) Act 1986 and with permission from the University of Edinburgh Animal Welfare Ethical Review Body. All sampling was carried out in accordance with UK Home Office regulations under Project Licence PP4825594.

Faecal samples are collected rectally at capture in August or, where a rectal sample could not be obtained, from observed defecation within several days of capture. As GIN eggs cannot be distinguished to species by eye, eggs from *Teladorsagia* spp., *Trichostrongylus* spp., *Chabertia ovina, Bunostomum trigonocephalum* and *Strongyloides papillosus* (hereafter referred to as strongyles) are combined and counted as one FEC using a modification of the McMaster technique (Wilson et al., 2004). Actual strongyle burden and FEC are strongly correlated, with lambs having higher parasite burdens than adults (Wilson et al., 2004). We used August FEC data collected from 1988 to 2017 from lambs (Males: *N* = 870; Females: *N* = 891), yearlings (Males: *N* = 356; Females: *N* = 510), and adults (Males: *N* = 547; Females: *N* = 2336) where associated August weight and annual home range data were available. Sample sizes indicated here are unique individual‐year samples. For individuals with multiple FEC data points for the same year, we kept the earliest dated sample and removed all others. We also removed FEC values greater than 5000 to minimise overdispersion (3 data points removed) resulting in 1081 individual adults with 2883 total observations, 866 yearlings, and 1761 lambs. Unbalanced sample sizes between sexes and ages are due to higher mortality in males and younger individuals resulting in fewer samples, as well as greater dispersal in males (Clutton‐Brock et al., 2004).

### 2.2 Vegetation and plant functional traits

The study area contains approximately 30% of the island's total sheep population (Clutton‐Brock et al., 1992). The vegetation of the study area is dominated by *Holcus lanatus*–*Agrostis capillaris* (*HA*) grassland on free draining, fertile soils, particularly around the village, *Molinia caerulea*‐dominated grassland on areas receiving drainage, and *Calluna vulgaris‐*dominated heath (wet and dry) on the steeper slopes around the bay (Jewell et al., 1974; Figure S1). HA grassland has the highest live standing‐crop biomass of the area (Crawley et al., 2004, 2021) and is preferred by Soay sheep (Jones et al., 2006). Wet *Calluna* heathland is rarely grazed even in years of resource limitation due to high sheep population numbers (Crawley et al., 2021). Vegetation surveys were conducted from 2008 to 2012 by visually estimating the proportion of species cover for every hectare (*N* = 267) to the nearest 5% with all estimates done by one observer. Species composition of the vegetation community remained relatively constant between 1993 and 2012 (summarised in Crawley, 2017). Regan et al. (2016) details further how botanical data was assessed and calibrated.

Trait values for all plant species were obtained from UK calibrated Ellenberg values (F and N) (Hill et al., 1999) and the LEDA Traitbase (CanHt, SLA, LeafSize and LDMC; Kleyer et al., 2008). While data from such databases do not account for intraspecific variation, it is the most practical method to quantify vegetation functional traits over such a large study area (Pakeman & Fielding, 2020). We chose four plant functional traits and two measures of plant habitat preferences to characterise the vegetation (Table 1). These traits were chosen for their potential relevance to parasite transmission or disease susceptibility, for example, due to increased digestibility of vegetation for grazers or more suitable microclimates for strongyle larvae. LDMC, SLA and Ellenberg nitrogen content (N) can all be used as proxies for preference/digestibility, as sheep tend to favour vegetation which are lacking fibrous compounds, easy to digest, and with high nutritional content (Gardarin et al., 2014; Pakeman, 2014). Canopy height (CanHt), Ellenberg moisture value (F) and leaf size (LeafSize) could be associated with enhancing parasite survival in their free‐living stages with high values for each indicating favourable microclimate conditions (Table 1). Community‐weighted means (CWM) were calculated for each hectare of the study area by multiplying the matrix of chosen functional trait values by species with the matrix of species by site abundance using the FD package v.1.0–12 (Laliberté et al., 2014; Laliberté & Legendre, 2010). CWM values have a strong theoretical base (Grime, 1998) and have been used successfully to address the linkages between management and vegetation, vegetation and ecosystem function/services, and across trophic levels (Lavorel et al., 2013). The range of CWMs for CanHt, LeafSize and F were spread across the study area. Low LDMC, high SLA, and high N were concentrated around the former settlement in the Village Bay area (Figure S1).

**TABLE 1:** Definitions and predicted effects of plant functional traits used.

Term	Description	Predicted effect on GIN transmission/survival	Predicted effect on sheep condition
CanHt	Distance between the base of a plant and its highest photosynthetic tissue (m).^a^	Positive: more favourable microclimate conditions	Positive: greater nutrition for given bite size
LDMC	Ratio of dry mass to fresh mass (mg/g).^b^	Negative: less palatable vegetation reducing host density	Negative: reduced palatability at high values, greater nutrition at low values
Leaf Size	Area of one side of a fresh leaf (mm^2^).^a^	Positive: more favourable microclimate conditions	Positive: greater nutrition for given bite size
SLA	Area of one side of a fresh leaf divided by the dry weight (mm^2^/mg).^a^	Positive: more palatable vegetation increasing host density	Positive: increased palatability at high values
F	Ellenberg moisture indicator (on a scale of 1–9). Low values indicate very low soil moisture content.^c^	Positive: more favourable microclimate conditions	—
N	Ellenberg nitrogen indicator (on a scale of 1–9). Low values indicate highly infertile sites.^c^	Positive: more palatable vegetation increasing host density	Positive: greater nutrition

^a^ Weiher et al. (1999).

^b^ Wilson et al. (1999).

^c^ Hill et al. (1999).

**FIGURE 2:**
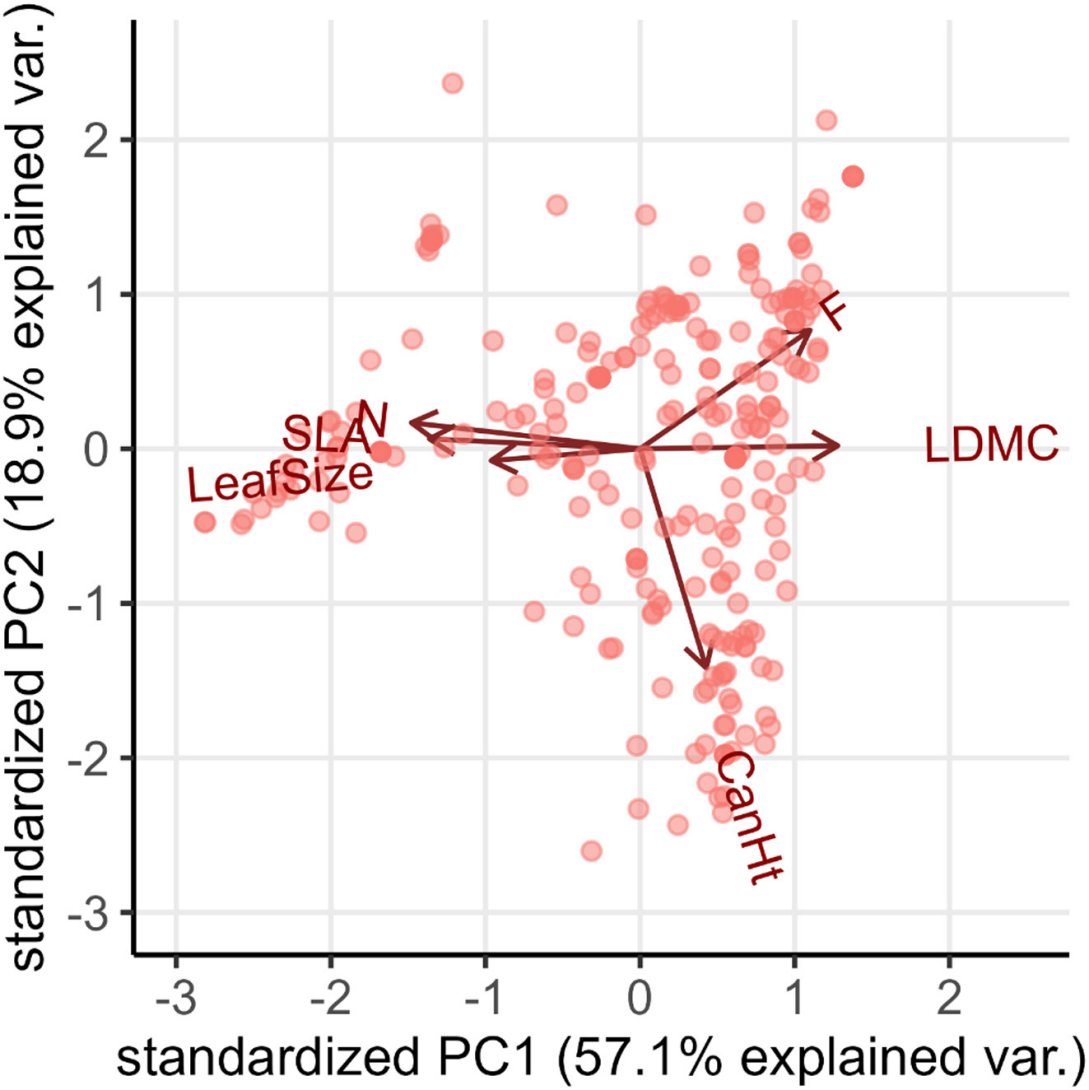
Principal component analysis (PCA) of functional trait values calculated for each hectare of the study area. The total variance explained by the first two axes is 76.0%. The loading scores and contributions of each variable are given in Table S2.

To account for correlations between functional traits (Table S1), we ran a principal component analysis using the stats package within R (R Core Team, 2021) to generate a combined measure of grazing preference. This resulted in a first principal axis of variation (PC1) explaining 57.1% of trait variance (Figure 2) with strong loadings in one direction from LMDC and in the opposite direction from LeafSize, SLA and N (Figure 2, Table S2). PC1 is consistent with expected sheep dietary preferences, with high values of PC1 indicating tougher to digest and less nutritious vegetation, and we chose to include it as a single variable in subsequent analysis. CanHt and F both had higher loadings on PC2 than PC1, but they were almost orthogonal to each other, and so were used in future models as separate terms rather than using the aggregated PC2. F also had a relatively high loading on PC1 and was correlated to the variable associated with PC1, indicating some confounding between F and PC1 was possible; wetter areas were also characterised by tougher, harder to digest leaf material. However, we kept F and PC1 as separate terms for further analysis because they describe traits that may influence disease dynamics differently. For instance, PC1 incorporates vegetation traits that are more likely to influence sheep nutritional intake through dietary preferences whereas F is more likely to influence strongyle egg and larval survival.

### 2.3 Home range quality estimation

In all, 30 study area censuses are carried out each year (10 each in spring, summer, and autumn). During each census, all individuals seen along a set route are noted, along with their location to the nearest 100 m × 100 m Ordnance Survey grid square. Adult Soay sheep tend to segregate into social groups according to sex, with females exhibiting greater philopatry to their birth area than males. While male juveniles leave their mothers at approximately 6 months old, females remain in the vicinity of their mother until about 2 years old (Clutton‐Brock et al., 2004).

We calculated home ranges based on census sightings from 1988 to 2017; years for which FEC data were available. We used the R package adehabitathr v.0.4.19 (Calenge, 2006) to estimate home ranges. Coordinates for census points are recorded as the most south‐westerly corner of a pre‐determined hectare, so we corrected the coordinates to give the centre of each hectare (easting +50 m, northing +50 m). As many observations have identical hectare references, we added a random number between −20 and 20 (a jitter of up to 20 m) to both easting and northing coordinates of each census observation to prevent errors with kernel estimates (Regan et al., 2016). To maximise home range accuracy, we used all observations for an individual with at least 10 census observations in a year (Number of individuals per year with home range and parasite data: median: 200; range: 28–294). We used a 70% isopleth to calculate the core home range (Powell, 2000; Regan et al., 2016) and overlaid the core home range onto the vegetation hectare squares previously developed. To account for different contributions from each hectare square to home ranges, we used proportional weighting of the number of observations of every individual in each square. This resulted in a mean value for each functional trait for each individual sheep's home range.

### 2.4 Statistical analysis

We conducted all statistical analyses with August FEC as our response variable in R v.4.0.4 (R Core Team, 2021). Individuals were categorised as adults, yearlings or lambs due to the pronounced variation in FEC across the age groups (Sweeny et al., 2022). Spatial autocorrelation, where samples or individuals closer together in time and space are more alike than those further apart, is becoming increasingly important to consider in studies of disease ecology (Albery et al., 2022). To quantify spatial autocorrelation, we included a stochastic partial differentiation equation (SPDE) random effect in Integrated Nested Laplace Approximation (INLA) models from the inla package v.21.02.23 (Lindgren et al., 2011; Rue et al., 2009). The SPDE spatial field represents how the response variable is structured across the study system. For more detail on the mathematical basis of SPDE, see Lindgren et al. (2011). For more detail on the use of INLA and SPDE in a similar study system of red deer, see Albery et al. (2019). To do so, we first developed a separate spatial mesh for adults, yearlings and lambs constrained by the coastline boundaries. Each age‐specific mesh is made up of the average coordinate for each individual‐year home range and allows interpolation of spatially distinct samples. Any average coordinate that fell in the ocean was removed (*N* = 6). All subsequent models were run using the age‐appropriate mesh.

The base model for all age categories included fixed effects of sex and August weight, and year as a random effect. SPDE (spatial field) was added to models separately as a random effect. The model for the adult age group also included age as a continuous covariate to account for immunosenescence, and individual identity as a random effect to account for pseudo‐replication. All models were fitted with negative binomial distributions. To base+SPDE models for each age group, we added vegetation traits – PC1, CanHt and F – separately. If the δDIC was greater than 2, we added the other vegetation traits individually and tested model fit by comparing DIC values at each iteration. We identified the best fitting model as the one with the fewest parameters within 2 DIC units of the model with lowest overall DIC.

## 3 RESULTS

**FIGURE 3:**
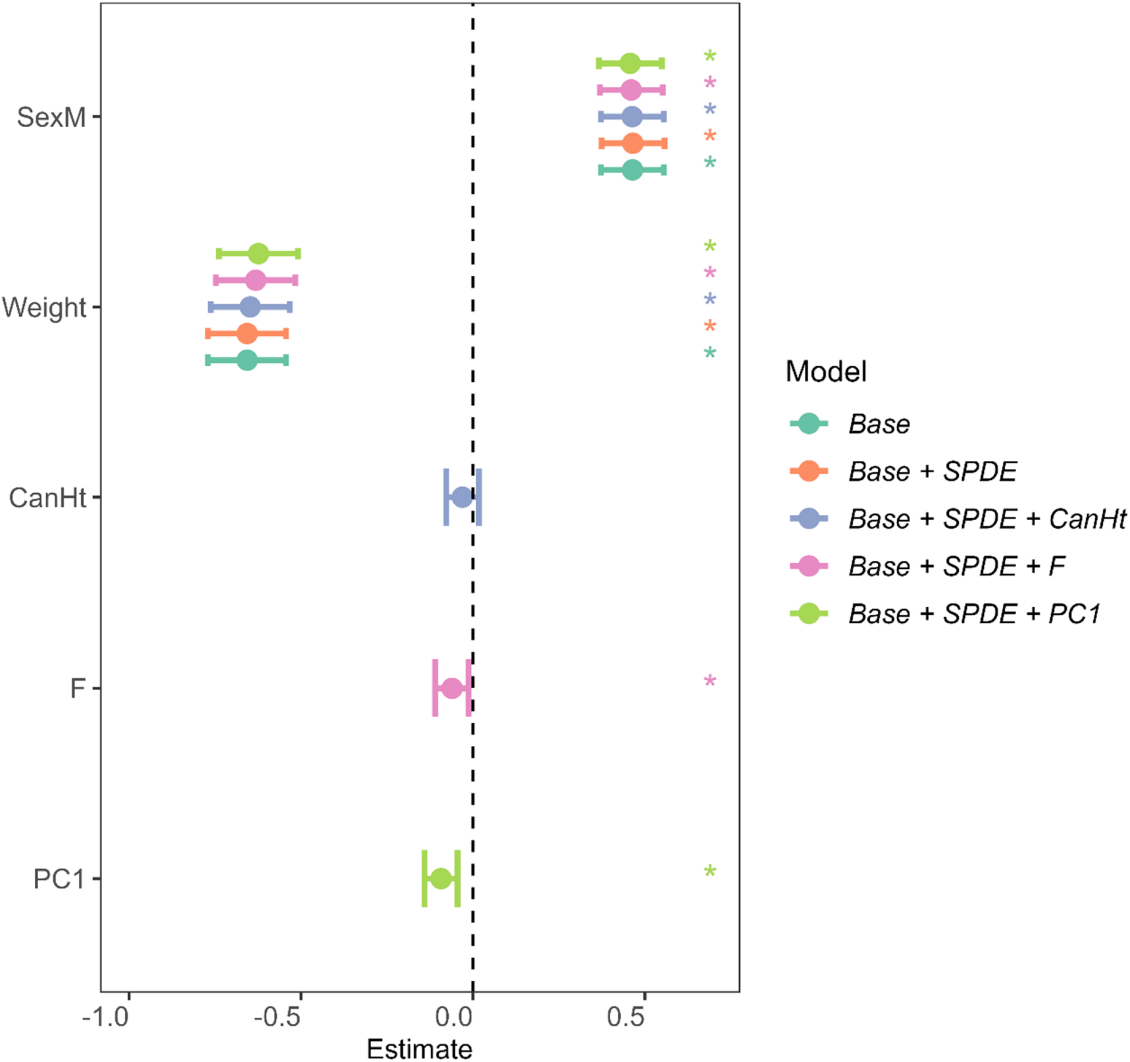
Confidence intervals (95%) for models on lambs. Base model includes sex and weight as fixed effects, and year as a random effect. The similarity in confidence intervals of each term across models suggests that the effects are consistent following inclusion of SPDE. Each model, and the combination of terms included therein, is assigned a colour and, as not all models include all terms, not all models can be compared for a given term. Traits are significant in the model if the confidence interval does not cross the central dashed line (significance indicated by asterisks). Positive estimates indicate a positive effect of the term on strongyle burdens.

**FIGURE 4:**
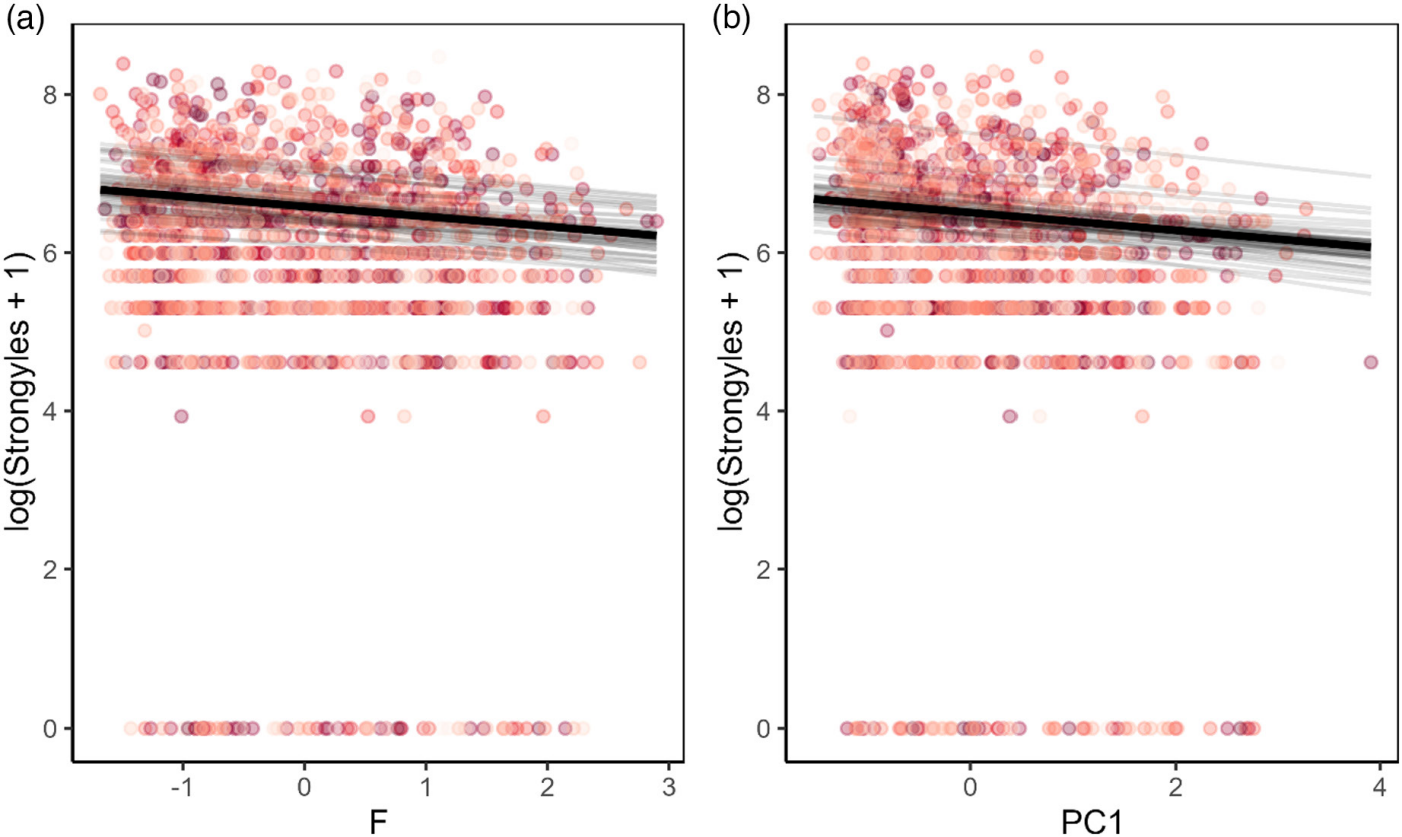
Effect of vegetation functional traits (a) F and (b) PC1 on FEC in lambs from final model outputs. The functional trait values are standardised around 0 and show a significant decrease in strongyle burdens as trait values increased. F: Mean: −0.125; 95% confidence interval: (−0.213, −0.047); *p* = 0.00124. PC1: Mean: −0.11; 95% confidence interval: (−0.171, −0.052); *p* = 0.00023.

**FIGURE 5:**
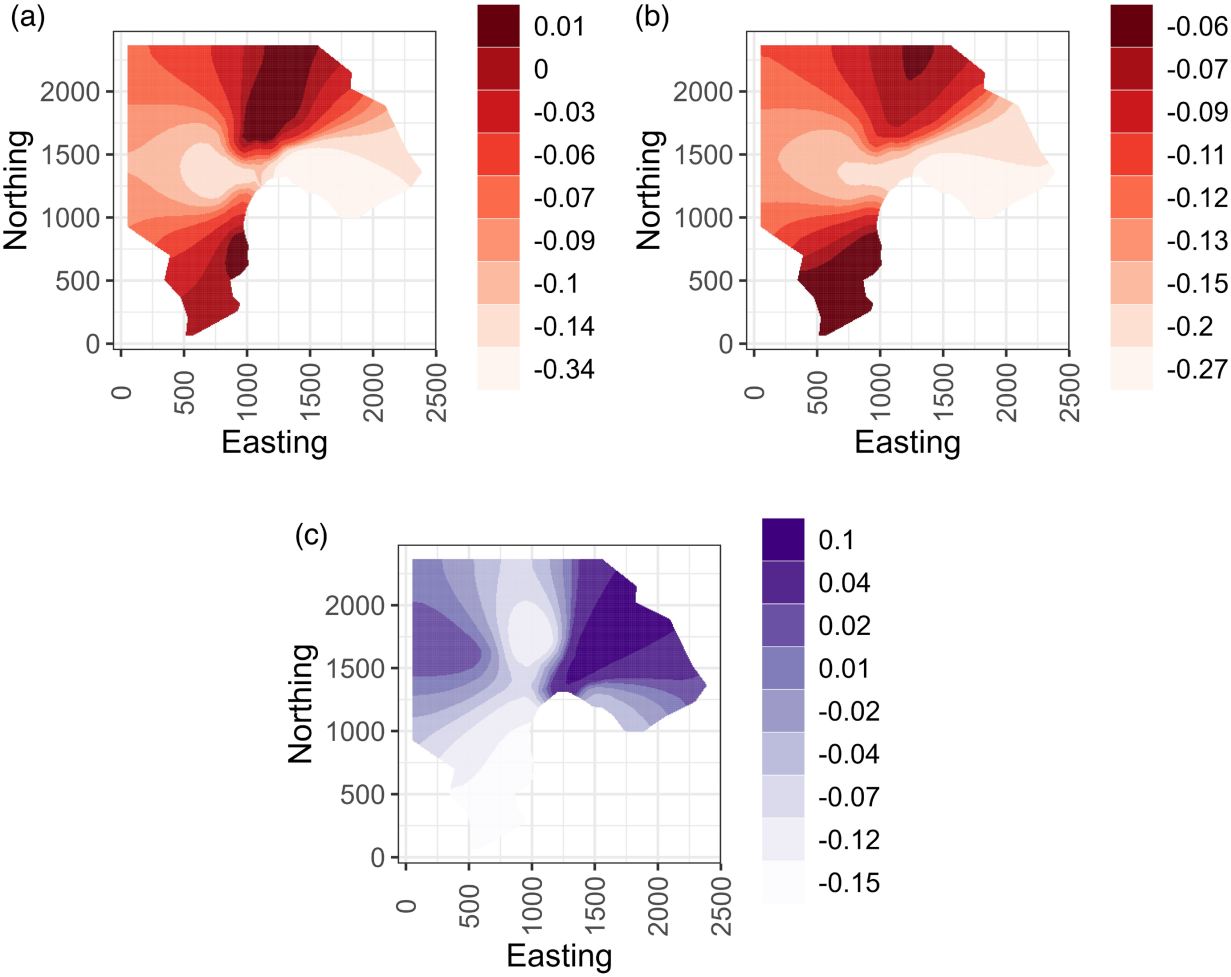
Spatial fields for lamb and adult FEC models. Lamb spatial fields models include base terms and functional traits (a) F or (b) PC1. Adult spatial field model (c) includes base terms. Colour scale shows the lower bounds of the spatial effect quantiles with darker colours indicating higher parasite counts. Eastings and northings are standardised coordinates (in units of 1 m) from the southern‐ and western‐most points of the study area and the spatial fields cover the Village Bay area (see Figure 1).

We found evidence for effects of spatial autocorrelation in August FEC for lambs and adults, but not for yearlings (δDIC: adults: 2.53; yearlings: −1.18; lambs: 4.27; Table 2). In lambs, we found additional evidence for effects of vegetation functional traits beyond spatial structuring of FEC, with higher values of F and of PC1 (high LDMC and low N, SLA, and LeafSize) associated with lower FEC (Table 2, Figure 3). In contrast, the best supported model in terms of DIC for yearlings and adults did not include any of the vegetation traits (adults: Base+SPDE; yearlings: Base; Table 2). As expected, males had higher FEC than females and weight was negatively associated with FEC across age groups, although there was no effect of continuous age within the adult age group (Figure 3, Figure S2). For lambs, inclusion of the spatial effect improved model fit (δDIC: 4.27, Table 2) and the addition of either F or PC1 to the SPDE model provided further improvement (δDIC: F: 13.03; PC1: 13.63, Table 2). Estimates from these models revealed significant negative effects of both F and PC1 when included individually in separate FEC models (Figures 3 and 4). However, including both F or PC1 in the same FEC model provided less explanatory power compared to models including just one of these terms (δDIC: −0.92 versus model including only F and −1.52 versus model including only PC1, Table 2). This suggests the two vegetation scores were explaining largely the same variance in FEC. For example, both high F and high PC1 could be associated with lower FEC in lambs due to shared effects of these two vegetation scores on parasite transmission or host condition (see Section 4). Examination of the spatial field of the lamb models including F or PC1, which would illustrate spatial autocorrelation in FEC after accounting for effects of the vegetation traits, weight and sex, suggest higher strongyle densities in the north and south of the study area (Figure 5a,b). Adult spatial fields suggest high strongyle densities in the north‐east of the study area (Figure 5c).

**TABLE 2:** DIC values of model construction. Models are presented in sequential order of term addition. Base models include sex, weight and year for all age categories, and age and individual ID for adults only. Terms were retained in the model if DIC values decreased by >2 δDIC. Final models (indicated in bold) were chosen based on DIC values (>2 δDIC) and least‐term parsimony.

Age category	Model	DIC	δDIC
Adult	Base	27611.71	0.00
	**Base+SPDE**	**27609.18**	**2.53**
	Base+SPDE+CanHt	27612.07	−0.36
	Base+SPDE+F	27611.74	−0.03
	Base+SPDE+PC1	27613.63	−1.92
Yearling	**Base**	**11245.89**	**0.00**
	Base+SPDE	11247.07	−1.18
	Base+SPDE+CanHt	11249.06	−3.17
	Base+SPDE+F	11249.92	−4.03
	Base+SPDE+PC1	11249.34	−3.45
Lamb	Base	26315.04	0.00
	Base+SPDE	26310.77	4.27
	Base+SPDE+CanHt	26309.92	5.12
	**Base+SPDE+F**	**26302.01**	**13.03**
	**Base+SPDE+PC1**	**26301.41**	**13.63**
	Base+SPDE+CanHt+F	26303.36	11.68
	Base+SPDE+CanHt+PC1	26303.35	11.69
	Base+SPDE+F+PC1	26302.93	12.11

## 4 DISCUSSION

We found age‐dependent effects of vegetation traits after controlling for spatial autocorrelation on strongyle burdens in Soay sheep. In lambs, vegetation traits (F and PC1) were negatively associated with strongyle burdens. Strongyle burdens in adults and yearlings showed no association with vegetation. Yearlings were also the only age category where accounting for spatial autocorrelation did not significantly improve the base model fit, with high parasite burdens in the north‐east, and in the north and south of the Village Bay area for adults and lambs respectively. Our findings confirm the importance of fine‐scale spatial variation in the landscape ecology of disease in natural systems and offer a new approach to understanding the relationship between habitat and infection status for field studies of herbivores. Importantly, we show that effects of habitat on host–parasite systems can vary among host demographic groups and highlight the importance of understanding this among‐host variation for disease ecology.

### 4.1 Parasite associations with PC1 and F in lambs

High values of PC1 (high LDMC and low N, SLA, and LeafSize) reflect low preference vegetation with low digestibility. The effects of PC1 on lamb strongyle burdens were as expected: higher strongyle burdens were associated with more digestible vegetation traits. It is unlikely that this is a condition‐mediated effect as we included weight in our models—weight has consistently been shown as a good predictor of individual condition in this study system (Jones et al., 2005; Milner et al., 1999)—and areas of good grazing should then be associated with more resources for immunity and lower FEC. This suggests that exposure to infective larval stages could be more important in determining parasite burdens than individual condition. While experimental work in wood mice *Apodemus sylvaticus* showed increased resistance to helminth infection following diet supplementation (Sweeny, Clerc, et al., 2021), nutrition effects can operate through both exposure and susceptibility processes and produce various outcomes for disease prevalence (Becker & Hall, 2014). In Soay sheep, much of the variation in parasite burdens is likely down to landscape heterogeneity with sheep density driving greater parasite exposure and transmission between individuals. However, since we controlled for spatial autocorrelation in our models, it is likely that there are further mechanisms behind transmission than solely spatial location.

One theory could be that social grouping and dynamics play a larger role than spatial location. Soay sheep do show some sex‐based social segregation; males tend to form a dominance hierarchy, and male lambs tend to leave their mothers earlier than female lambs (Clutton‐Brock et al., 2004). Additionally, adult males have a different seasonal FEC pattern to lambs or adult females, potentially leading to altered transmission and infection dynamics (Sweeny et al., 2022). Incorporating social networks and accounting for how different interactions (e.g. mother–offspring relationships) may drive disease transmission could provide further information behind the mechanisms of such transmission. Previous work on the European badger *Meles meles* provides a framework for such work by comparing spatial models with social interaction models to determine which best described parasite distribution (Albery et al., 2020).

We found that high levels of moisture (F) were associated with lower parasite burdens. While contrary to our expectations—rainfall has been found to favour strongyle larval survival (Zouyed et al., 2016)—moisture may not be limiting strongyle survival on St. Kilda due to its hyper‐oceanic climate. That F and PC1 explain largely the same variance in our models could be due to them both being similarly correlated to a causal factor driving FEC burdens. For example, sheep may avoid the wettest areas (Putfarken et al., 2008) or areas of less preferred vegetation (Catorci et al., 2014), thus reducing rates of host density‐dependent transmission and leading to a negative association between moisture levels, or low preference vegetation, and strongyle burdens. There may also be some unknown influence of fine‐scale spatial heterogeneity of the plant community on parasite burdens (Johnson et al., 2016). We used averaged vegetation traits over the entirety of an individual's home range and so can only provide a limited picture of the heterogeneity of vegetation available to an individual. Sheep are selective about their food sources (Illius et al., 1995), so simply because their home range has a particular trait value does not mean the plants contributing to this will be consumed. Additionally, greater spatial heterogeneity tends to increase community stability in both theoretical models (Briggs & Hoopes, 2004; Murdoch, 1977), empirical predator–prey systems (Daugherty, 2011; Mitsunaga & Fujii, 1997) and host–parasite interactions (Brockhurst et al., 2006). Therefore, small‐scale structural and environmental variation in habitat must be considered along with large‐scale generalisations of habitat quality in studies of wildlife disease and ecology.

### 4.2 Age‐specific vegetation–parasite associations

We found support for effects of vegetation traits on parasite counts beyond spatial structuring in lambs only. There was some evidence for spatial structuring of FEC in adults but little support for either vegetation effects or spatial effects in yearlings. To our knowledge, this is the first evidence that fine‐scale habitat effects on parasite burdens might vary across demographic groups in the wild. This age‐specific effect of space and vegetation on strongyle burdens may be driven by differences in foraging behaviour between age classes. Young, domesticated cattle learn to graze faster when in the presence of experienced individuals (Costa et al., 2016), and moose *Alces alces* calves learn to identify toxic plants from their mother (Edwards, 1976). Additionally, as the vegetation trait values used in this study are average values and do not consider intraspecies variation, it may be that adults are more adept at identifying the variation that minimises any nutrition‐parasite trade‐off. For example, Soay sheep preferentially graze shorter plants with a comparatively reduced parasite risk, over taller plants associated with greater nutritional intake (Hutchings et al., 2002). As lambs may not yet have developed learned behaviours regarding grazing, this suggests that they may not have learnt to avoid vegetation with higher parasite transmission risk. Therefore, their parasite burdens may be more correlated with functional traits at the scale of this investigation than adults, who are more selective consumers due to these learned behaviours. As lambs are dependent on their mother and tend follow her, there could be a potential conflict between adult survival and performance from feeding on the best vegetation, and exposure of their offspring to higher parasite burdens. Indeed, Soay sheep lambs are less selective in consuming vegetation and are less avoidant of areas of high larvae count than adults (Hutchings et al., 2002).

Lambs are also immunologically naïve and less resilient to infection than adults (Nussey et al., 2012). Soay sheep rapidly acquire resistance to GINs, with yearlings having higher antibody levels and lower FEC than lambs (Sparks et al., 2018). As yearlings are less susceptible to disease than lambs, this may contribute to the lack of spatial structure in yearling FEC. It is also likely that the yearling age group has less variation in individual condition as weak lambs will be less likely to survive their first winter and mature into yearlings, and yearlings are not affected by immunosenescence as in adults. This suggests that strongyle burdens in older individuals are largely driven by variation in immune‐mediated resistance or tolerance and condition, while spatial variation of lamb burdens may be more directly linked to eggs ingested from pasture and so more easily discerned. Therefore, including measures of immune response in Soay sheep may provide further insight into whether it is a lack of immune response in lambs or other factors driving this difference between age classes.

### 4.3 Wider implications and conclusions

We present a novel approach for analysing how the interaction between individuals and their environment influences disease dynamics. While incorporating the vegetation of an individual's home range into analyses of fitness traits has been done previously in the Soay sheep (Regan et al., 2016), using vegetation functional traits to summarise an individual's home range allows for more generalisable conclusions about processes across study systems. In addition, there is growing awareness that fine‐scale spatial variation is an important driver of parasite dynamics in natural systems (Albery et al., 2021; Becker et al., 2020), to which our findings lend further support. Unfortunately, we cannot separate effects of exposure linked to sheep density from effects of exposure linked to vegetation quality using this approach. This could be addressed in future using a combination of small‐scale, short‐term studies investigating the effect of plant species on pasture larvae counts in the field and models capable of extrapolating such data to estimate landscape‐level exposure over longer time periods (e.g. Garcia‐Méndez et al., 2022; Rose et al., 2015). The effect of PC1 is consistent with high‐quality grazing areas being more heavily used by sheep and thus higher strongyle burdens in lambs. This means that patterns in strongyle burdens are related to selective grazing patterns and transmission. However, we cannot exclude that this effect may be due to more specific characteristics of the vegetation itself and how this impacts larval development and transmission.

In conclusion, we present fine‐scale patterns of variation in parasite burden, which are relatively rarely explored in wild systems (Albery et al., 2022). Our models suggest there are effects of vegetation type on strongyle burdens beyond spatial structuring of parasite burdens which may well be driven by preferential grazing and local density. However, we also show that these patterns are not present in all members of population and may depend on various demographic factors such as age, immunological susceptibility or condition. This has implications for our understanding of wildlife epidemiology and health as not all individuals or demographic groups experience the same disease risk. In addition, identifying spatial hot spots for parasite transmission could lead to more effective grazing management plans to reduce disease‐related costs and dependence on anthelmintics within livestock systems.

## AUTHOR CONTRIBUTIONS

Ellis Wiersma, Amy R. Sweeny, Daniel H. Nussey, and Robin J. Pakeman conceived of and designed the study. Ellis Wiersma and Amy R. Sweeny conducted the analyses. Ellis Wiersma, Amy R. Sweeny, Daniel H. Nussey, and Robin J. Pakeman prepared the first draft of the manuscript, and all authors contributed to revised drafts. Jill G. Pilkington, Xavier Bal, and Josephine M. Pemberton led the fieldwork on St Kilda and JGP collected most of the faecal egg count data.

## CONFLICT OF INTEREST STATEMENT

The authors declare no conflicts of interest.

## Supporting information


**Figure S1.** Vegetation rasters of each vegetation functional trait showing spatial variation in values within the study area. Vegetation rasters were generated from the community weighted means of each grid square of the study area. Each grid square is 100 m^2^. For all traits, higher values are green and lower values are light pink. Eastings and northings are standardized coordinates (in units of 1 m) from the southern‐ and western‐most points of the study area.
**Figure S2.** Confidence intervals (95%) for models of (a) adults and (b) yearlings. Base model includes sex, and weight as fixed effects, and year as a random effect. Base models for adults also include individual as a random effect and age as a continuous fixed effect. The similarity in confidence intervals of each term across models suggests that the effects are consistent following inclusion of SDPE. Each model, and the combination of terms included therein, is assigned a colour and, as not all models include all terms, not all models can be compared for a given term. Traits are significant in the model if the confidence interval does not cross the central dashed line (significance indicated by asterisks). Significant traits are indicated by asterisks. Positive estimates indicate a positive effect of the term on FEC.
**Table S1.** Correlation matrix of the vegetation variables used.
**Table S2.** Summary of PCA with (a) loadings from PCA and (b) contributions of each variable to dimensions in PCA.

## Data Availability

Data are available from the Dryad Digital Repository at https://doi.org/10.5061/dryad.z08kprrjf (Wiersma et al., 2023).
