# Supplementary material for: Age‐specific impacts of vegetation functional traits on gastrointestinal nematode parasite burdens in a large herbivore
Source: J Anim Ecol. 2023 Jul 5;92(9):1869–80. doi: 10.1111/1365-2656.13978 (PMC10952545; doi:10.1111/1365-2656.13978)
Supplement: Supplementary file 1 — Figure S1. Vegetation rasters of each vegetation functional trait showing spatial variation in values within the study area. Vegetation rasters were generated from the community weighted means of each grid square of the study area. Each grid square is 100 m2. For all traits, higher values are green and lower values are light pink. Eastings and northings are standardized coordinates (in units of 1 m) from the southern‐ and western‐most points of the study area. Figure S2. Confidence intervals (95%) for models of (a) adults and (b) yearlings. Base model includes sex, and weight as fixed effects, and year as a random effect. Base models for adults also include individual as a random effect and age as a continuous fixed effect. The similarity in confidence intervals of each term across models suggests that the effects are consistent following inclusion of SDPE. Each model, and the combination of terms included therein, is assigned a colour and, as not all models include all terms, not all models can be compared for a given term. Traits are significant in the model if the confidence interval does not cross the central dashed line (significance indicated by asterisks). Significant traits are indicated by asterisks. Positive estimates indicate a positive effect of the term on FEC. Table S1. Correlation matrix of the vegetation variables used. Table S2. Summary of PCA with (a) loadings from PCA and (b) contributions of each variable to dimensions in PCA. [file JANE-92-1869-s001.docx]

**SUPPLEMENTARY MATERIAL**

***Supplementary Figure S1.*** *Vegetation rasters of each vegetation functional trait showing spatial variation in values within the study area. Vegetation rasters were generated from the community weighted means of each grid square of the study area. Each grid square is 100 m^2^. For all traits, higher values are green and lower values are light pink. Eastings and northings are standardized coordinates (in units of 1 m) from the southern- and western-most points of the study area.*

***
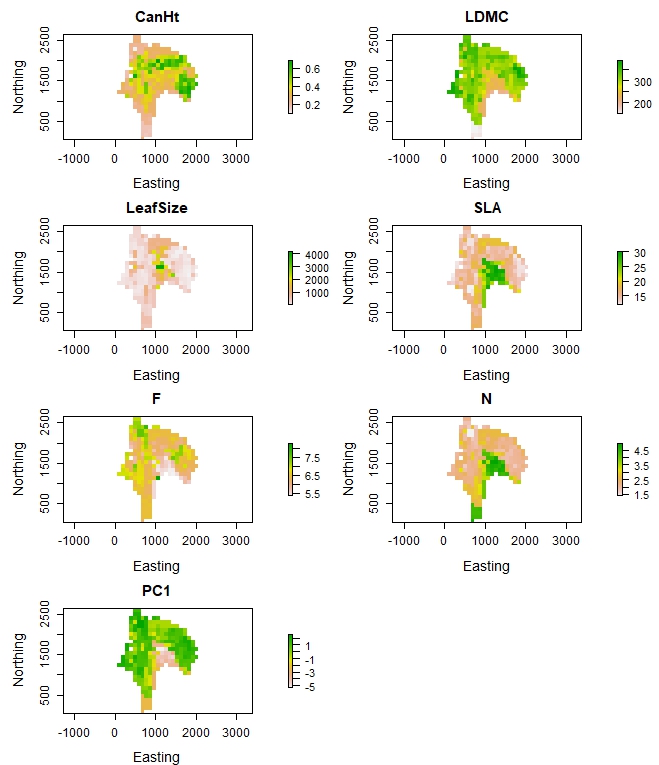
***

***Supplementary Table S1.*** *Correlation matrix of the vegetation variables used.*

|  | **CanHt** | **LDMC** | **LeafSize** | **SLA** | **F** | **N** |
| --- | --- | --- | --- | --- | --- | --- |
| **CanHt** | 1 | - | - | - | - | - |
| **LDMC** | 0.18156 | 1 | - | - | - | - |
| **LeafSize** | -0.075734 | -0.33605 | 1 | - | - | - |
| **SLA** | -0.25039 | -0.63020 | 0.56965 | 1 | - | - |
| **F** | -0.15115 | 0.54239 | -0.30515 | -0.56025 | 1 | - |
| **N** | 0.37592 | -0.84074 | 0.51618 | -0.82990 | -0.66138 | 1 |

***Supplementary Table S2****. Summary of PCA with a) loadings from PCA and b) contributions of each variable to dimensions in PCA.*

1. Loadings from PCA

|  | **PC1** | **PC2** | **PC3** | **PC4** | **PC5** | **PC6** |
| --- | --- | --- | --- | --- | --- | --- |
| **CanHt** | 0.15107 | -0.87277 | 0.10067 | -0.29668 | 0.25308 | 0.23077 |
| **LDMC** | 0.45192 | 0.012363 | 0.35674 | 0.71506 | 0.12689 | 0.37543 |
| **LeafSize** | -0.34070 | -0.046988 | 0.86318 | -0.10560 | -0.34214 | -0.091766 |
| **SLA** | -0.48179 | 0.039663 | 0.13561 | 0.20654 | 0.81108 | -0.21773 |
| **F** | 0.38846 | 0.47284 | 0.28175 | -0.58837 | 0.37552 | 0.24280 |
| **N** | -0.52331 | 0.103797 | -0.14055 | -0.026296 | -0.062573 | 0.83127 |

1. Contributions from PCA

|  | **PC1** | **PC2** | **PC3** | **PC4** | **PC5** | **PC6** |
| --- | --- | --- | --- | --- | --- | --- |
| **CanHt** | 2.2822 | 76.172 | 1.0135 | 8.8021 | 6.4049 | 5.3253 |
| **LDMC** | 20.423 | 0.015285 | 12.726 | 51.130 | 1.6102 | 14.095 |
| **LeafSize** | 11.608 | 0.22079 | 74.508 | 1.1151 | 11.706 | 0.84209 |
| **SLA** | 23.213 | 0.15731 | 1.8390 | 4.2657 | 65.786 | 4.7408 |
| **F** | 15.090 | 22.357 | 7.9383 | 34.617 | 14.102 | 5.8951 |
| **N** | 27.385 | 1.0774 | 1.9754 | 0.069147 | 0.39154 | 69.102 |

***Supplementary Figure S2. Confidence intervals (95%) for models of (a) adults and (b) yearlings****. Base model includes sex, and weight as fixed effects, and year as a random effect. Base models for adults also include individual as a random effect and age as a continuous fixed effect. The similarity in confidence intervals of each term across models suggests that the effects are consistent following inclusion of SDPE. Each model, and the combination of terms included therein, is assigned a colour and, as not all models include all terms, not all models can be compared for a given term. Traits are significant in the model if the confidence interval does not cross the central dashed line* *(significance indicated by asterisks). Significant traits are indicated by asterisks. Positive estimates indicate a positive effect of the term on FEC.*

*
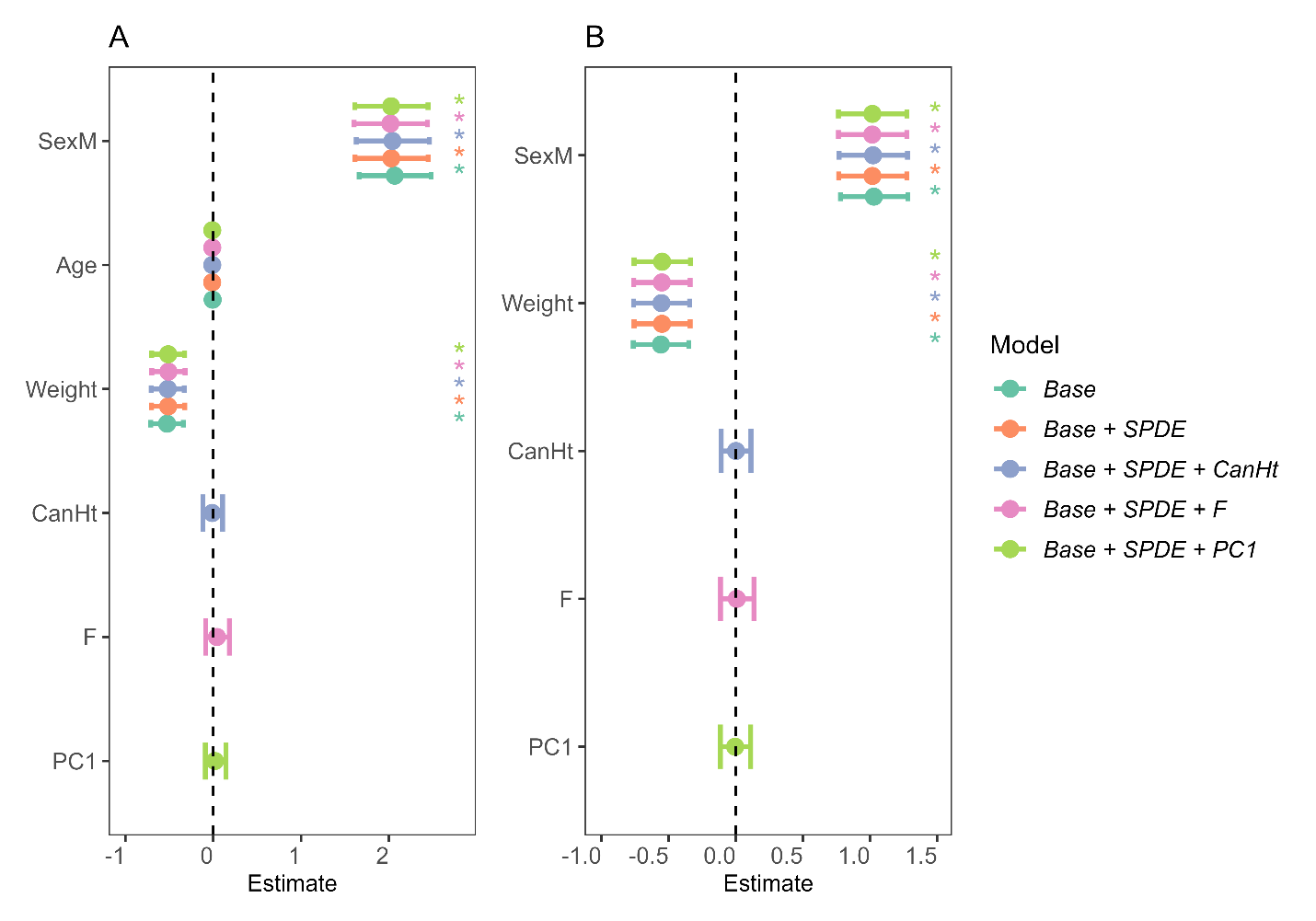
*
